# Assessment of Heavy Metal Contamination in the Surrounding Soils and Surface Sediments in Xiawangang River, Qingshuitang District

**DOI:** 10.1371/journal.pone.0071176

**Published:** 2013-08-08

**Authors:** Min Jiang, Guangming Zeng, Chang Zhang, Xiaoying Ma, Ming Chen, Jiachao Zhang, Lunhui Lu, Qian Yu, Langping Hu, Lifeng Liu

**Affiliations:** 1 College of Environmental Science and Engineering, Hunan University, Changsha, China; 2 Key Laboratory of Environmental Biology and Pollution Control (Hunan University), Ministry of Education, Changsha, China; 3 Department of Civil and Environmental Engineering, University of Waterloo, Waterloo, Canada; 4 College of Resources and Environment, Hunan Agricultural University, Changsha, China; University of Illinois at Chicago, United States of America

## Abstract

Xiawanggang River region is considered to be one of the most polluted areas in China due to its huge amount discharge of pollutants and accumulation for years. As it is one branch of Xiang River and the area downstream is Changsha city, the capital of Hunan Province, the ecological niche of Xiawangang River is very important. The pollution treatment in this area was emphasized in the Twelfth Five-Year Plan of Chinese government for Xiang River Water Environmental Pollution Control. In order to assess the heavy metal pollution and provide the base information in this region for The Twelfth Five-Year Plan, contents and fractions of four heavy metals (Cd, Cu, Pb and Zn) covering both sediments and soils were analyzed to study their contamination state. Three different indexes were applied to assess the pollution extent. The results showed this area was severely polluted by the four heavy metals, and the total concentrations exceeded the Chinese environmental quality standard for soil, grade III, especially for Cd. Moreover, Cd, rated as being in high risk, had a high mobility as its great contents of exchangeable and carbonates fractions in spite of its relative low content. Regression analysis revealed clay could well explain the regression equation for Cd, Cu and Zn while pH and sand could significantly interpret the regression equation for Pb. Moreover, there was a significant correlation between Non-residual fraction and *I_geo_* for all the four metals. Correlation analysis showed four metals maybe had similar pollution sources.

## Introduction

Environmental contamination by heavy metals is a serious and worldwide problem that accompany with the rapid industrialization and urbanization in many countries. It is noticed that human-induced metals like Pb, Hg and Cu have been detected in both Greenland and Antarctica snow samples that were remote from human beings [1]–[5]. Sediments/soils are not only basic components of our environment as they provide nutrients for living organisms, but also serve as reservoirs for deleterious chemical species which cause negative effects on aquatic system and human health [6]–[8]. It is now widely recognized that the measurement of total metal concentration in sediments/soils is not sufficient to provide information about the exact dimension of pollution by heavy metals [9]. The environmental behavior of heavy metals critically depends on their specific chemical forms and on their binding state (precipitated with primary or secondary minerals, complexed by organic ligands, etc.), which influence their bioavailability, mobility, and toxicity to organisms [10]–[12]. Thus, there is considerable interest in improving the understanding of element-solid phase association in natural and polluted systems.

Qingshuitang District, which is located in Zhuzhou City of Hunan Province, is a typical heavy industrial base specially in smelting and chemical in China. Due to industrial structure and historical reasons, its regional environmental pollution is very severe making it one of the most serious areas of national environmental issues. As one branch of Xiang River, Xiawangang River accounts for most of industrial wastewater and part of domestic sewage of Qingshuitang area. In 2006, owing to improper construction of dredging engineering of Xiawangang River and inappropriate preventive measures, Xiawangang River section to Changsha section of Xiang River was severely polluted by Cd, resulting in water contamination of the source of Xiangtan and Changsha water works. The pollution treatment of Xiawangang River was specially stressed in The Twelfth Five-Year Plan of Chinese government for Xiang River Water Environmental Pollution Control. However, information regarding the heavy metals pollution in this area is limited. In order to assess the real heavy metal contamination status of surface sediments and surrounding soils of Xiawangang River, we have carried out an investigation in this region. To achieve a comprehensive assessment of the impacts of heavy metals, different indexes including geo-accumulation index (*I_geo_*), risk assessment code (*RAC*), Ratio pollution index (*RPI*) were applied. The main objective of this study is to get more information on the heavy metal pollution status in this area and provide guidance for dredging and remediation projects for The Twelfth Five-Year Plan of Chinese government.

## Materials and Methods

### 2.1. Sample Collecting and Processing

**Figure 1:**
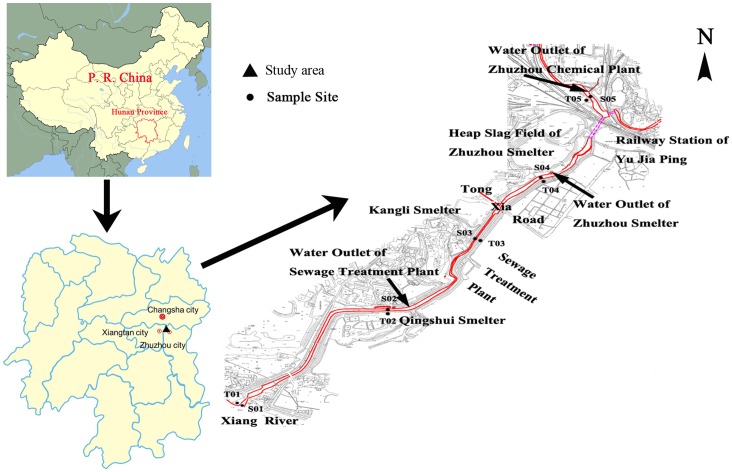
Study area and geographical location of ten stations in Xiawangang River.

The location of the sampling sites is shown in Fig. 1. Considering the representativeness of the pollution in Xiawangang River, five sample sites were chosen near the outlets for discharging sewage of the industry companies, and each site included one soil and sediment respectively. A total of ten surface samples (five sediments and five soils) were collected with a clean polymethyl methacrylate shovel and a small brush, and three subsamples nearby were collected and then mixed thoroughly to obtain a bulk sample for each site. In order to get the accordant samples and to compare the results of the experiment, all the samples were collected under the same condition in one day. The collected samples were kept in polyethylene ziploc bags and preserved under freezing condition (<−10°C) before processing. All these samples were air dried at room temperature and sieved through a 2 mm nylon sieve to remove big coarse debris. The samples were then rubbing with a pestle and mortar, and sieved through a 0.149 mm nylon sieve before use. The surface water samples at the site of the sediment were also collected in polyethylene bottles for physicochemical parameters and metals. The heavy metal water samples were collected in bottles by acidification in the field with concentrated HNO_3_.The bottles were kept in ice cake on the way to the laboratory, and then stored in fridge at 4°C before analysis.

No specific permits were required for the described field studies. The studying area is not privately-owned or protected in any way and the field studies did not involve endangered or protected species.

### 2.2. Analytical Methods

The pH of water samples was determined in the field. Carbonate, total alkalinity, sulphate, chloride and phosphate were detected in laboratory [13].

The pH of soil/sediment samples was measured in the ratio of 2.5 (w:v = sample: distilled water) with a pH glass electrode. A small portion of the sample was ignited by muffle for 4 h at 550°C. The losses during bakeout and ignition were determined separately as indirect index of organic matter content (OM) [14]. Other parameters including sand-silt-clay, bulk density (BD), pore space (PS) and organic carbon (OC) were also detected by reference [15].

For the total heavy metal content detection, 0.1 g samples were picked by a high precision analytical balance. Subsequently, the samples were placed in Teflon tubes and digested with HNO_3_, HF, and HClO_4_. Then the solutions were diluted with 2% (v/v) HNO_3_ to a final volume of 50 mL, and analyzed for Cd, Cu, Zn, Pb by an atomic absorption spectrophotometer (AAnalyst700, Perkin-Elmer Inc, US).

Sequential extraction was performed by the five-stage Tessier method, which is widely applied in various studies of heavy metal [16]–[19]. The details of the Tessier method used in this study had been described elsewhere [20].

### 2.3. Quality Control

The analytical data quality was guaranteed by quality assurance and quality control methods, including the use of standard operating procedures, reagent blanks, and three sub-samples determination through the implementation of laboratory. The relative standard deviations (%RSDs) of the sub-samples were <10%, indicating excellent reproducibility of the equipment and operation procedures. The results of five fractions were summed up and compared with total concentration to check the recovery, and the percentage recoveries of heavy metals varied from 85.29% to 103.31%.

### 2.4. Assessment of Pollution

#### 2.4.1. Geo-accumulation Index (*I_geo_*)

As defined by Müller [21], the geo-accumulation index is a quantitative measure of metal pollution. This assessment index was cited by studies in soils and sediments [22], [23]. *I_geo_* values are calculated using the following mathematical formula:
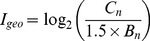
(1)


Where *C_n_* is the measured content of element, and *B_n_* is the background or pristine value of the element. The constant factor 1.5 is the background matrix correction factor due to lithogenic effects. The classification of the contamination degree according to the *I_geo_* values is listed in Table 1.

**Table 1:** Pollution grades of geo-accumulation index of the metals.

Classification	*I_geo_*	Pollution status
0	*I_geo_* <0	Unpolluted (UP)
1	0< *I_geo_* ≤1	Unpolluted to moderately polluted (UMP)
2	1< *I_geo_* ≤2	Moderately polluted (MP)
3	2< *I_geo_* ≤3	Moderately to strongly polluted (MSP)
4	3< *I_geo_* ≤4	Strongly polluted (SP)
5	4< *I_geo_* ≤5	Strongly to extremely polluted (SEP)
6	*I_geo_* >5	Extremely polluted (EP)

#### 2.4.2. Risk Assessment Code (*RAC*)

Assessment of *RAC*, based on the strength of the bond between metals and other components in soil or sediment, also considers the ability of metals to be released and enter into the food chain [24]. Therefore, *RAC* can give a clear indication of soil or sediment reactivity, which in turn assesses the risk connected with the presence of heavy metals in environment. *RAC* assesses the availability of metals by applying a scale to the percentage of metal in the carbonate and exchangeable fractions. These fractions are weakly bound metals which could equilibrate with the aqueous phase and thus become more rapidly bioavailable [25], [26]. When the percentage of the carbonate and exchangeable fractions is less than 1%, there is no risk (NR). For a range of 1–10%, there is low risk (LR), medium risk (MR) for a range of 11–30%, high risk (HR) for 31–50% and very high risk (VHR) for 51–100% [25], [27].

#### 2.4.3. Ratio Pollution Index (*RPI*)

The *RPI* (ratio pollution index) was the ratio of heavy metal concentrations and their background values, and can be defined by the following equation:
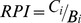
(2)


Where *C_i_* represents the measured concentration of the element *i*, and *B_i_* is the the geochemical background value of the element. It reflected the heavy metal pollution state by human activities.

## Results and Discussion

### 3.1. Physicochemical Characteristics and Heavy Metal Concentrations of the Water

The Physicochemical parameters and heavy metal concentrations of Xiawangang River were showed in Table 2. The pH range of Xiawangang River was 7.72–8.34, indicating the moderately alkaline nature. The chloride varied from 261.6 mg/L to 618.5 mg/L. The values were much higher than the Xiang River (12.6 mg/L). The Carbonate contents were low, which ranged from 8.3 mg/L to 11.6 mg/L (site 02 was not detected). Total alkalinity varied between 156.9 mg/L and 291.8 mg/L, which were higher than Xiang River (92.2 mg/L). It also revealed that hydroxyl and bicarbonate radical were the main factors in total alkalinity compared with carbonate. The total concentration of sulphate in Xiawangang River varied from 37.1 mg/L to 436.2 mg/L. This was fairly high and could be attributed to the use of sulfuric acid by the surrounding factories. Phosphate in Xiawangang River was observed from 0.06 mg/L to 0.18 mg/L, which was similar with Xiang River. And it was in the scope of Integrated Wastewater Discharge Standard (GB 8978–1996). In water samples, the concentration of Cu varied from 0.31 mg/L to 0.82 mg/L, which was less than the reference value (GB 8978–1996), while the other metals were found to be in the range of Cd 0.03–0.32, Pb 0.64–1.21 and Zn 2.79–5.69, all on mg/L unit.

**Table 2:** The main characteristics and heavy metal concentrations in Xiawangang River.

Site	pH	Carbonate^d^	Total alkalinity^d^	Sulphate^d^	Chloride^d^	Phosphate^d^	Cd^d^	Cu^d^	Pb^d^	Zn^d^
01^a^	7.72	8.3	227.1	401.6	522.2	0.16	0.32	0.82	0.93	4.33
02^a^	7.76	ND^c^	291.8	436.2	357.9	0.18	0.15	0.62	0.82	3.82
03^a^	8.34	9.9	156.9	218.9	261.6	0.07	0.08	0.75	0.64	2.79
04^a^	7.93	16.5	214.7	188.3	618.5	0.09	0.07	0.31	1.21	5.47
05^a^	8.25	11.6	273.9	37.1	473.6	0.06	0.03	0.43	0.97	5.69
Xiang River^b^	7.52	ND^c^	92.2	17.8	12.6	0.11	0.001	0.005	0.004	0.02
Reference value^e^	6–9	–	–	–	–	1.0	0.1	1.0	1.0	5.0

a Surface water at the site of sediment.

b Surface water at the site of Xiang River (Zhuzhou section).

c Not detected (ND).

d Concentration (mg/L).

e Integrated Wastewater Discharge Standard (GB 8978–1996).

### 3.2. Physicochemical Characteristics and Heavy Metal Concentrations of Soils and Sediments

pH, organic matter (OM), particle size distribution (sand-silt-clay), bulk density (BD), pore space (PS) and organic carbon (OC) were measured to get the general physicochemical characteristics of sediments/soils in this study. As shown in Table 3, pH of the studied sites all showed alkalescency. This might be explained by the waste water discharge into the river which contained various alkaline matters (e.g. ammoniate). The organic matter content in soil was 7.50% to 11.63%, and 4.16% to 11.36% in sediment. Their difference among the samples from sediments and soils was insignificant. However, obvious difference was observed in sand between sediment and soil. Sand was found to be dominant in sediment samples (50.71%–62.59%) followed by silt (19.89%–29.05) and clay (13.45%–29.40%). This may be the result of continuous deposition of alluvium on the riverbed in Xiawangang River. The results were similar with the study by Singh et al. [28]. The percentage of sand in soil samples was 25.46% to 42.62% followed by silt (33.90%–45.80%) and clay (19.34%–39.51%). BD in soils ranged from 1.15 g/cm^3^ to 1.20 g/cm^3^, while the value in sediments was 1.27 g/cm^3^ to 1.38 g/cm^3^. PS was closely associated with BD. PS was 18.44% to 24.61% in sediment, while it was nearly 50% in soil. High percentage of pore in soil of Xiawangang River resulted in a very loose texture. The content of organic carbon varied from 42.64 g/kg to 72.29 g/kg in soils, 21.90 g/kg to 59.70 g/kg in sediments, respectively.

**Table 3:** The main characteristics and heavy metal concentrations in soil and sediment samples from Xiawangang River.

	Site	pH	OM^a^	Sand(%)	Silt(%)	Clay(%)	BD^b^	PS^c^	OC^d^	Cd^e^	Cu^e^	Pb^e^	Zn^e^
Soil	T01	7.69	10.40	35.71	35.63	28.66	1.20	48.90	52.41	220.8±9.7	425.8±13.2	762.3±9.4	4842.1±131.3
	T02	8.12	11.18	25.46	39.15	35.39	1.15	50.32	72.29	512.1±13.8	864.1±19.0	4472.7±61.6	14105.5±378.7
	T03	7.86	11.63	26.59	33.90	39.51	1.16	49.18	57.61	499.9±19.9	920.5±27.1	5146.3±74.5	12491.2±549.2
	T04	8.04	7.50	42.62	38.04	19.34	1.20	49.01	42.64	53.5±9.0	460.3±18.7	2213.9±75.4	3734.4±57.8
	T05	7.66	10.21	32.80	45.80	21.40	1.17	48.82	60.32	56.9±10.8	469.0±16.4	1629.2±49.8	5615.5±76.5
													
Sediment	N01	8.41	4.16	62.59	23.64	13.77	1.31	23.31	21.90	50.2±9.3	213.9±11.8	308.2±11.3	2139.9±65.0
	N02	8.13	9.19	50.71	19.89	29.40	1.27	18.44	51.48	112.0±7.5	464.7±8.1	1050.0±42.8	4110.3±140.0
	N03	7.86	11.36	54.70	26.04	19.26	1.32	20.72	59.70	173.1±14.5	323.1±18.4	344.0±9.2	5075.8±127.8
	N04	8.19	4.64	57.50	29.05	13.45	1.38	24.61	31.37	96.0±11.8	454.7±13.4	712.5±10.2	2989.9±173.9
	N05	8.04	7.27	60.65	20.57	18.78	1.37	21.90	33.75	13.8±4.2	358.9±8.9	616.2±7.6	1898.1±154.0
RV^f^	National standard-Grade I^g^									0.02	35	35	100
	National standard- Grade II^g^									0.6	100	350	300
	National standard- Grade III^g^									1.0	400	500	500
	Background of Hunan Province									0.079	25.4	27.3	88.6

a Organic matter content (OM) (%).

b Bulk density (BD) (g/cm^3^).

c Pore space (PS) (%).

d Organic carbon (OC) (g kg^-1^).

e Heavy metal concentration (µg g^-1^). Results are expressed as the mean ± standard deviation.

f Reference value (RV).

g Environment quality standard for soils in china (National Environment Protection Agency of China, 1995). Grade I was mainly suitable for the soil of nature reserve, sources of drinking water, tea plantation, pasture, and other protected areas; Grade II was mainly applicable to the soil of general farmland, vegetable and Orchard; Grade III primarily suitable for the soil of woodland, and the soil of high background values which had high concentration of pollutants, also including the farmland near the mine.

**Figure 2:**
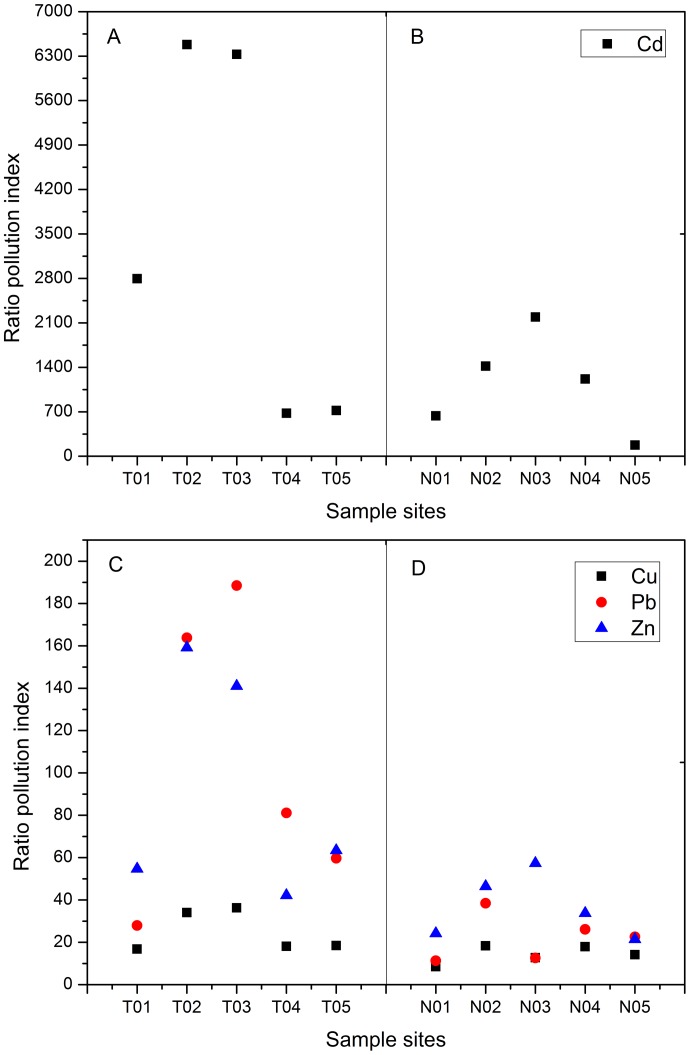
Ratio pollution index of Cd, Cu, Pb and Zn in sediment and soil samples from Xiawangang River.

The total concentrations of heavy metals and corresponding reference values were also shown in Table 3. The levels of investigated metals varied from 13.8 to 512.1 µg g^-1^ for Cd, 213.9 to 920.5µg g^-1^ for Cu, 308.2 to 5146.3µg g^-1^ for Pb, and 1898.1 to 14105.5µg g^-1^ for Zn, respectively. The highest concentrations of Cd, Cu, Pb and Zn were respectively about 512.1, 2.3, 10.3 and 28.2 times higher than the value of Chinese environmental quality standard for soil, grade III. Overall, the spatial variations of the concentrations of Cd, Cu, Pb and Zn in soils were more significant than that in sediments. For the comparison purpose, Fig. 2 showed the ratio pollution index (*RPI*) of heavy metal concentrations with their background values from Hunan Province soils. Obviously, the highest contamination metal was Cd. *RPI* values of Cd were all above 600 except for site N05. *RPI* values for Cu, Pb and Zn were 8.42–36.4, 11.29–188.51, and 21.42–140.98, respectively. Unlike other metals in the sample sites, the Cd, Cu, Pb and Zn concentrations in soils, and Cd and Zn concentrations in sediments showed a first rise after reducing trend. The spatial distributions of Cu and Pb concentrations in sediment were unregularly. Except for Cd at site T04 and N04, the total contents of heavy metals were clearly higher in soils than associated sediments, which was different from the study of Lake Victoria [29]. It may be that the movement of water washed out the top sediments which resulted in a higher concentration in soils than in sediments.

The metal concentrations of Xiawangang River were compared with the published date of other rivers (Table 4). The results revealed that the soils and sediments of Xiawangang River were severely polluted by the four metals, especially for Cd and Zn. The extent of metal contamination in Xiawangang River was much more serious than other rivers at home and abroad (Table 4).

**Table 4:** Comparison the metal concentrations of Xiawangang River with the other rivers (BDL is below detection limit).

Location	Metal concentration/µg g^-1^				References
	Cd	Cu	Pb	Zn	
Xiawangang River, sediment, China	13.8–173.1	213.9–464.7	308.2–1050.0	1898.1–5075.8	This study
Xiawangang River, soil, China	53.5–512.1	425.8–920.5	762.3–5146.3	3734.4–14105.5	This study
Tigris River, sediment, Turkey	0.7–4.9	11.2–5075.6	62.3–566.6	60.1–2396	[30]
Gomti River, sediment, India	0.34–8.38	BDL-35.03	6.27–75.33	3.06–101.73	[28]
Hindon River, sediment, India	BDL-11.80	0.85–282.25	12.00–380.50	14.50–404.50	[31]
Dommel River, soil, Netherlands	0.72–10.9	5.79–39.6	–	48.3–310	[32]
Solofrana river valley, soil, Italy	–	70–565	21–98	72–135	[33]
Luan River, sediment, China	0.03–0.37	6.47–178.61	8.65–38.29	21.09–25.66	[34]
Shing River, sediment, Hong Kong	22–47	207–1660	126–345	32–2200	[35]

### 3.3. Speciation of Heavy Metal

Metal speciation analysis, as proposed by Tessier, et al. [36], has been used to obtain the following five fractions: exchangeable (F1); bound to carbonates (F2); bound to Fe-Mn oxides (F3); bound to organic matter (F4); residual (F5). The mobility of heavy metals generally decreases in the order of extraction sequence i.e. F1> F2> F3> F4> F5. The first two fractions (F1 and F2) are considered to be weakly bounded metals which may equilibrate with the aqueous phase and thus become more rapidly bioavailable [28]. The Fe-Mn oxide and organic matter fractions can provide a sink for heavy metal. These fractions will most likely be affected and may be transformed into F1 or F2 by the redox potential and pH [25]. Therefore, the potential of their eco-toxicity should be not ignored. The residual fraction is steady and strongly bound in the crystal minerals and, consequently, has low mobility.

**Figure 3:**
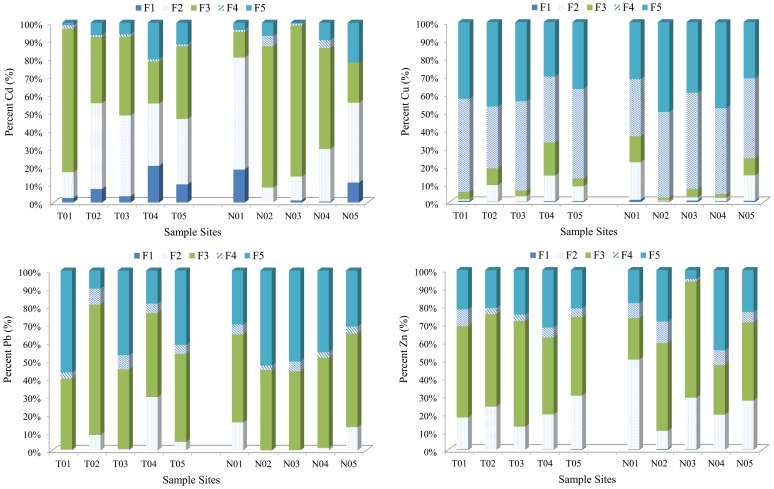
Fractionation of Cd, Cu, Pb and Zn in sediment and soil samples from Xiawangang River. F1: exchangeable, F2: bound to carbonates, F3: bound to Fe/Mn oxides, F4: bound to organic matter, F5: residual.

Fig. 3 showed the percentages of heavy metal concentrations that were extracted in each step of the sequential extraction procedure used in the study. Cd was mainly bound to F2 and F3 (approximate 80% with even contribution). The relative proportions of Cd in F4 and F5 were generally very low as compared to other metals. The former two fractions (F1 and F2), having direct toxicity to environment, accounted for 44.43% and 37.77% in soil and sediment with even contribution, respectively. Especially, N01 had the highest F1 and F2 (close to 80.61% with even contribution) among all the sites. This might be explained by the fact that Cd had special affinity for clay mineral structure due to its ionic radii and tended to combine with carbonate minerals at high pH [37], [38]. The results suggested that Cd was the most labile metal because of its stronger affinity to non-residual fraction. Although the mean total amount of Cd was lower than that of other metals, the amount poured into river and plant should be managed.

Cu was predominantly associated with F4 and F5 (44.70% and 40.03% with even contribution, respectively) both in soil and sediment, however single F4/F5 didn’t occupy very big proportion in total content. The percentage of Cu associated with different fractions was in the order: F4> F5> F3> F2> F1. Our finding is in similar with the result obtained by Li, et al. [39]. A few researchers have reported that a high concentration of Cu was significantly associated with organic matter in sediments [40], [41], and some researchers have also found that Cu showed a tendency towards the organic phase, as it formed strong association with oxygen and sulphur atoms in soils [42]–[44]. However, Cu is considered more readily soluble. When the environment conditions change (such as pH, drying and oxidation) it would be released from associations with organic matter [45].

Pb mainly existed in F3 and F5 (49.17% and 39.36% with even contribution, respectively). Especially, T01 provided the highest percentage of F5 (56.64%) while T02 provided the highest percentage of F3 (72.34%). Luo, et al. [46] reported that F3 and F5 were the main fractions in branch sediment of Poyang Lake. Akcay, et al. [6] also found that Pb was mainly associated with F3 and F5 in Buyak Menderes and Gediz river sediments. Pb bound to exchangeable fraction was not detected either in soil or sediment, and low percentage of Pb was also found well below 9% with even contribution in carbonate fraction and organic fraction both in soil and sediment. However, Pb should be managed seriously for its potential ecotoxicity considering the high percentage in F3 when the environment condition changed [47].

The major fraction of Zn was associated with F3 with an average of 49.50% in soil and 41.50% in sediment, respectively. Compared to F3, a relatively high percentage of about 24% with even contribution was bound to residual fraction both in soil and sediment. Meanwhile, a very low percentage of Zn was found well below 1% in exchangeable fraction. The proportion of Zn associated with organic fraction was similar with that of Pb (below 8%). These results also indicated that Zn had great potential ecotoxicity and bioavailability to the environment. Moreover, as Zn has a large total concentration, its environment risk will be more serious.

### 3.4. Assessment of Heavy Metal Pollution

#### 3.4.1. Assessment of geo-accumulation index (*I_geo_*)

The geo-accumulation index (*I_geo_*) was used to evaluate the heavy metal pollution by comparing current concentrations with reference value (Background of Hunan Province). The results were shown in Table 5. The *I_geo_* values of soil samples in this study were 8.82–12.08 for Cd, 3.48–4.59 for Cu, 4.22–6.97 for Pb and 4.81–6.73 for Zn, respectively. In sediment samples, the *I_geo_* values were in the range of 6.87–10.51 for Cd, 2.49–3.61 for Cu, 2.91–4.68 for Pb and 3.84–5.26 for Zn, respectively. The results showed that Xiawangang River was severely polluted by investigated heavy metals, especially, all of the *I_geo_* values for Cd in both soil and sediment were above 5, meaning extremely polluted (EP). In terms of soil, the mean *I_geo_* values for Cu, Pb and Zn were 3.96, 5.81 and 5.94, respectively. It was implied that Pb and Zn also extremely polluted soil, while Cu strongly polluted soil. Compared with *I_geo_* values in soil, the values of sediment were a little lower. The mean *I_geo_* values were 3.20 (Cu), 3.74 (Pb) and 4.51 (Zn), respectively, suggesting that Cu moderately-strongly polluted sediments and that Pb polluted sediments strongly; meanwhile, Zn strongly-extremely polluted sediments. On the whole, Cd, Cu, Pb and Zn polluted both soil and sediment heavily, especially Cd. According to the mean *I_geo_* values, contamination levels of heavy metals were in the increasing order of Cu<Zn <Pb<Cd in soils, while it is Cu <Pb<Zn<Cd in sediments.

**Table 5:** Heavy metal geo-accumulation index (*I_geo_*) in soil and sediment samples from Xiawangang River.

Site	*I_geo_*/Pollution status			
	Cd	Cu	Pb	Zn
T01	10.86/EP	3.48/SP	4.22/SEP	5.19/EP
T01	12.08/EP	4.50/SEP	6.77/EP	6.73/EP
T01	12.04/EP	4.59/SEP	6.97/EP	6.55/EP
T01	8.82/EP	3.59/SP	5.76/EP	4.81/SEP
T01	8.91/EP	3.62/SP	5.31/EP	5.40/EP
Mean	10.54/EP	3.96/SP	5.81/EP	5.74/EP
N01	8.73/EP	2.49/MSP	2.91/MSP	4.01/SEP
N01	9.88/EP	3.61/SP	4.68/SEP	4.95/SEP
N01	10.51/EP	3.08/SP	3.07/SP	5.26/EP
N01	9.66/EP	3.58/SP	4.12/SEP	4.49/SEP
N01	6.87/EP	3.24/SP	3.91/SP	3.84/SP
Mean	9.13/EP	3.20/SP	3.74/SP	4.51/SEP

#### 3.4.2. Assessment of Risk Assessment Code (*RAC*)

Table 6 showed the classification of samples according to *RAC*. It was found that the *RAC* value of Cd ranged from 16.90% to 55.25% with the mean value of 44.43% in soil; meanwhile it was 8.38%–80.61% with an average of 37.77% in sediment, which revealed that Cd was posing a high risk. Especially at site T02, T04, N01 and N05, the *RAC* value of Cd was greater than 50%, which showed very high risk. As the toxicity and availability of Cd, it can pose serious threat to the environment. It can be seen that the percentages of Cu associated with F1 and F2 had some similarity with Pb both in soil and sediment. Except T04, N01 and N05, the *RAC* value of Cu and Pb was less than 10%, showed low risk. The percentages of Zn associated with F1 and F2 which ranged from 12.89% to 30.11% with the mean 20.97% in soil, 10.59% to 50.27% with the mean 27.37% in sediment, respectively, revealed moderate risk.

**Table 6:** Risk assessment codes of heavy metals in soil and sediment samples from Xiawangang River.

Site	RAC/R			
	Cd	Cu	Pb	Zn
T01	16.90%/MR	1.49%/LR	0.42%/NR	18.01%/MR
T02	55.25%/VHR	9.50%/LR	8.62%/LR	24.07%/MR
T03	48.41%/HR	3.25%/LR	0.70%/LR	12.89%/MR
T04	55.10%/VHR	14.76%/MR	29.68%/MR	19.74%/MR
T05	46.46%/HR	8.79%/LR	4.80%/LR	30.11%/MR
Mean	44.43%/HR	7.56%/LR	8.84%/LR	20.97%/MR
N01	80.61%/VHR	22.16%/MR	15.52%/MR	50.27%/HR
N02	8.38%/LR	0.89%/LR	0%/NR	10.59%/MR
N03	14.41%/MR	2.67%/LR	0.15%/NR	29.01%/MR
N04	29.88%/MR	2.33%/LR	1.42%/LR	19.62%/MR
N05	55.56%/VHR	14.84%/MR	12.89%/MR	27.37%/MR
Mean	37.77%/HR	8.58%/LR	6.00%/LR	27.37%/MR

### 3.5. Multivariate Statistical Analyses

Heavy metals and soil/sediment parameters usually have complicated relationships among them [48]. To further investigate the relationship between metals and the characteristics in soil/sediment, regression analysis was performed by SPSS with stepwise method which was chose to optimize variables. The results were showed in Table 7 and Table 8. From Table 7, only variable clay entered in the regression equation for Cd, Cu and Zn while sand and pH entered in regression equation for Pb after performing with stepwise method. F values of regression were 22.159, 21.469, 15.497 and 20.443 for Cd, Cu, Pb and Zn, respectively, showed significant level for four heavy metals at 99% confidence level. Table 8 showed the regression coefficients of Cd, Cu and Zn were positive, suggesting the higher the clay content was, the greater the content of heavy metals was. Clay-sized particles are usually characterized by large specific surface area and internal porosity that may act as a potential contaminant immobilizer in the internal pore network [49]. However, the Pb content was controlled by pH (positive coefficient) and sand (negative coefficient). The regression equations of four metals were statistically significant. Correlation analysis was also used to assess possible co-contamination from similar sources. A very significant correlation was found between Cd, Cu, Pb, Zn (r = 0.890–0.962) at 99% confidence level from Table 9. The high correlations between heavy metals may reveal that the four metals had similar pollution sources [48].

**Table 7:** The results of the global test of regression analysis.

Model	R	R^2^	Adjusted R^2^	F	Sig.	Durbin-Watson
Stepwise (Cd)^a^	0.857	0.735	0.702	22.159**	0.002	2.466
Stepwise (Cu)^b^	0.854	0.729	0.695	21.469**	0.002	1.792
Stepwise (Pb)^c^	0.903	0.816	0.763	15.497**	0.003	1.523
Stepwise (Zn)^d^	0.848	0.719	0.684	20.443**	0.002	2.452

** *P*<0.01.

a Cd = Constant, Clay.

b Cu = Constant, Clay.

c Pb = Constant, Sand, pH.

d Zn = Constant, Clay.

**Table 8:** The results of regression coefficients and collinearity diagnosis.

	Independent Variable	Coefficients		t	Sig.	Tolerance	VIF
		Unstandardized Coefficients	Standardized Coefficients				
Stepwise (Cd) a	Constant	−241.814		−2.551*	0.034		
	Clay	17.603	0.857	4.707**	0.002	1.000	1.000
Stepwise (Cu) b	Constant	−18.144		−0.154	0.881		
	Clay	21.495	0.854	4.634**	0.002	1.000	1.000
Stepwise (Pb) c	Constant	−20290.647		−1.825	0.111		
	Sand	−132.603	−1.081	−5.500**	0.001	0.681	1.469
	pH	3496.802	0.470	2.393*	0.048	0.681	1.469
Stepwise (Zn) d	Constant	−3844.798		−1.717	0.124		
	Clay	399.442	0.848	4.521**	0.002	1.000	1.000

* *P*<0.05,

** *P*<0.01.

a Cd = Constant, Clay.

b Cu = Constant, Clay.

c Pb = Constant, Sand, pH.

d Zn = Constant, Clay.

**Table 9:** Pearson correlation coefficients of heavy metals (n = 10).

	Cd	Cu	Pb	Zn
Cd	1	0.890**	0.849**	0.957**
Cu		1	0.962**	0.931**
Pb			1	0.918**
Zn				1

** Correlation is significant at the 0.01 level (two-tailed).

**Figure 4:**
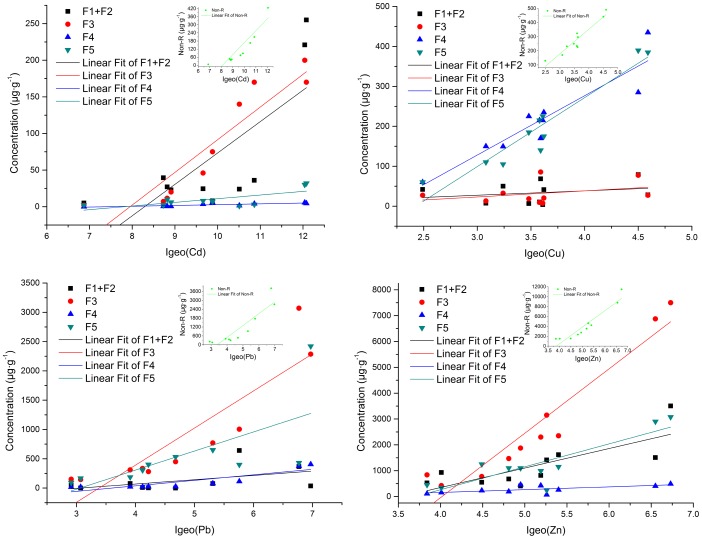
Relationships between the speciation concentrations and the corresponding *I_geo_*.

As discussed above, the environmental behavior of heavy metals critically depends on their speciation [50]. As we can see from Fig. 4, for Cd, there was a significant linear correlation between Fe/Mn oxides fraction (F3) and the corresponding *I_geo_* (R^2^ = 0.828). For Cu, organic matter fraction (F4) and residual fraction (F5) were significant correlation with *I_geo_* (R^2^ = 0.838, 0.898, respectively). For Pb, Fe/Mn oxides fraction (F3) and organic matter fraction (F4) were correlation with *I_geo_* (R^2^ = 0.770, 0.739, respectively). For Zn, only Fe/Mn oxides fraction (F3) was well correlation with *I_geo_* (R^2^ = 0.900). Moreover, an obvious correlation between Non-residual fraction (Non-R) and *I_geo_* was observed for all the four metals (R^2^ = 0.828 for Cd, R^2^ = 0.894 for Cu, R^2^ = 0.789 for Pb and R^2^ = 0.884 for Zn, respectively) compared with residual fraction. This indicated that human activities inputs were probably the major contribution for accumulation in sediments/soils of Xiawangang River [50].

### Conclusions

All of the investigated heavy metals have accumulated significantly both in soils and sediments in Xiawangang River. Zn and Pb were the most abundant elements with higher concentrations. Meanwhile, the concentration of four heavy metals was higher in soil samples nearby than that in sediment samples. The speciation data of metals suggested that Cd had a high availability in exchangeable and carbonate bound fractions. Cu was preferentially found in the organic and residual fraction, while Pb was mainly present in the Fe/Mn oxides and residual fraction. Zn was mostly bound to Fe/Mn oxides fraction. Contamination assessment based on *I_geo_* showed that Cd, Cu, Pb and Zn polluted both soil and sediment heavily, especially Cd both in soils and sediments, Pb in soils, and Zn in soils. According to *RAC*, Cd revealed high risk to the environment due to its high percentage of F1 and F2 in despite of its relative low content, while Cu and Pb showed low risk both in soils and sediments. In addition, Zn was classified as moderate risk.

The results of regression analysis revealed that clay was the main contribution and could well explain the regression equation for Cd, Cu and Zn, while pH and sand also significantly interpret the regression equation for Pb. The results of correlation analysis showed the four metals maybe had similar pollution sources such as human activities especially industrial inputs. There was an obvious linear correlation between Fe/Mn oxides fraction of Cd and *I_geo_*, between organic and residual fraction of Cu and *I_geo_*, between Fe/Mn oxides and organic fraction of Pb and *I_geo_*, and between Fe/Mn oxides fraction of Zn and *I_geo_*. This study also suggested the metal contamination cannot be simply evaluated by total concentration or single assessment alone. A complementary approach including sediment standard criteria, speciation, assessment of diffident methods and multivariate statistical analyses should be considered in order to provide a more accurate and comprehensive assessment of the risk of heavy metals to the environment.
